# Amplified Genes May Be Overexpressed, Unchanged, or Downregulated in Cervical Cancer Cell Lines

**DOI:** 10.1371/journal.pone.0032667

**Published:** 2012-03-07

**Authors:** Oscar Vazquez-Mena, Ingrid Medina-Martinez, Eligia Juárez-Torres, Valeria Barrón, Ana Espinosa, Nicolás Villegas-Sepulveda, Laura Gómez-Laguna, Karem Nieto-Martínez, Lorena Orozco, Edgar Roman-Basaure, Sergio Muñoz Cortez, Manuel Borges Ibañez, Carlos Venegas-Vega, Mariano Guardado-Estrada, Angélica Rangel-López, Susana Kofman, Jaime Berumen

**Affiliations:** 1 Unidad de Medicina Genómica, Facultad de Medicina, Hospital General de México, Universidad Nacional Autónoma de México, México, Distrito Federal, México; 2 Departamento de Biomedicina Molecular, Centro de Investigación y Estudios Avanzados del Instituto Politécnico Nacional, México, Distrito Federal, México; 3 Departamento de Genética, Facultad de Medicina, Hospital General de México, Universidad Nacional Autónoma de México, México, Distrito Federal, México; 4 Laboratorio de Genómica de Enfermedades Complejas, Instituto Nacional de Medicina Genómica, México, Distrito Federal, México; 5 Servicio de Oncología, Hospital General de México, México, Distrito Federal, México; 6 Servicio de Ginecología, Hospital General de México, México, Distrito Federal, México; 7 Unidad de Investigación Médica Enfermedades Nefrológicas, Centro Médico Nacional Siglo XXI, Instituto Mexicano del Seguro Social, México, Distrito Federal, México; 8 Departamento de Medicina Experimental, Facultad de Medicina, Universidad Nacional Autónoma de México, México, Distrito Federal, México; International Centre for Genetic Engineering and Biotechnology, Italy

## Abstract

Several copy number-altered regions (CNAs) have been identified in the genome of cervical cancer, notably, amplifications of 3q and 5p. However, the contribution of copy-number alterations to cervical carcinogenesis is unresolved because genome-wide there exists a lack of correlation between copy-number alterations and gene expression. In this study, we investigated whether CNAs in the cell lines CaLo, CaSki, HeLa, and SiHa were associated with changes in gene expression. On average, 19.2% of the cell-line genomes had CNAs. However, only 2.4% comprised minimal recurrent regions (MRRs) common to all the cell lines. Whereas 3q had limited common gains (13%), 5p was entirely duplicated recurrently. Genome-wide, only 15.6% of genes located in CNAs changed gene expression; in contrast, the rate in MRRs was up to 3 times this. Chr 5p was confirmed entirely amplified by FISH; however, maximum 33.5% of the explored genes in 5p were deregulated. In 3q, this rate was 13.4%. Even in 3q26, which had 5 MRRs and 38.7% recurrently gained SNPs, the rate was only 15.1%. Interestingly, up to 19% of deregulated genes in 5p and 73% in 3q26 were downregulated, suggesting additional factors were involved in gene repression. The deregulated genes in 3q and 5p occurred in clusters, suggesting local chromatin factors may also influence gene expression. In regions amplified discontinuously, downregulated genes increased steadily as the number of amplified SNPs increased (p<0.01, Spearman's correlation). Therefore, partial gene amplification may function in silencing gene expression. Additional genes in 1q, 3q and 5p could be involved in cervical carcinogenesis, specifically in apoptosis. These include *PARP1* in 1q, *TNFSF10* and *ECT2* in 3q *and CLPTM1L*, *AHRR*, *PDCD6*, and *DAP* in 5p. Overall, gene expression and copy-number profiles reveal factors other than gene dosage, like epigenetic or chromatin domains, may influence gene expression within the entirely amplified genome segments.

## Introduction

Cervical cancer (CC) is the second most common cancer in women worldwide, affecting 500,000 individuals each year, and it is the main cause of death of women with cancer in developing countries [1]. The viral oncoproteins E6 and E7 of the high-risk human papillomaviruses (HPV) play an important role in carcinogenesis. They inhibit various cellular targets, including the tumor-suppressor proteins p53 and pRB, disrupt key cellular processes, such as apoptosis and cell-cycle control, and lead to genomic instability and neoplastic development [2]. Despite the damage caused by the oncoviral proteins, CC is a rare complication of the viral infection because most infections are transient and do not evolve into neoplastic lesions. On average, it takes 12–15 years before a persistent HPV infection may, via the premalignant stages of cervical intraepithelial neoplastic lesions (CIN), lead to CC [3]. These findings suggest HPV infection alone does not cause the disease and other factors, such as abnormal host genes, could be associated with the development of invasive cancer. Several genomic regions have been identified with changes in the number of DNA copies (copy number-altered regions, CNAs) in CC through the analysis of the tumor genome by using methods such as comparative genomic hybridization (CGH), fluorescence in situ hybridization (FISH), and microarrays of SNPs. Gains in 1q, 3q, 5p, 8q, and 20q and deletions in 2q, 3p, 4p, 4q, 5q, 6q, 8p, 11q, 13q, 18q, and Xq have been frequently reported in both CC [4]–[10] and CC-derived cell lines [9], [11]–[16]. Genomic imbalances can contribute to deregulated expression of oncogenes and tumor suppressor genes in cancer cells, and the accumulation of such altered genes has been correlated with tumor progression [17]. However, the contribution of these alterations to cervical carcinogenesis is still a matter of debate. Gains of 3q [5]–[9], [18], [19] and 5p [5], [13], [20]–[22] are the most frequent chromosomal alteration in cervical carcinomas, and they have also been described in other solid tumors [23]–[25]. The smallest consensus region of 3q amplification in CC maps into chromosomal cytobands 3q26–27 [6]–[9], [14], [18], suggesting some genes located in these regions could be involved in cervical carcinogenesis. Some of them, including *TERC* [26], [27] and *PIK3CA* [28], are considered candidate oncogenes for CC. Large regions of 3q, including the loci where *TERC* and *PIK3CA* are located, have been confirmed amplified by FISH in the interphase nucleus of cervical tumors and metaphase Chr in cell lines [14], [29]. However, a detailed characterization of these amplified genes has not been performed and only the gain of *PIK3CA* has been validated by quantitative PCR [28]. It has not been demonstrated that *TERC* is upregulated in tumor samples or cell lines in which it is amplified, and the correlation between the amplification and upregulation of the *PIK3CA* gene is still controversial. Amplification of *PIK3CA* is not associated with increased gene expression in tumor samples [30], [31]. However, it has been associated with an increased amount of the protein by western blot in cell lines [28] and increased protein activity in tumors [32] and cell lines [28]. The extent to which these recurrent chromosomal alterations are relevant for tumor development is still largely unknown. On the other hand, the full amplification of 5p is well documented by FISH in tumor samples and cell lines [13], [16], [29]. Some amplified genes harbored by this region and proposed to be involved in CC, such as *SKP2*, *TERT*, *TRIO*, *RNASEN*, and *PRKAA1*, have been found to be upregulated in tumor samples [22] and cell lines [13], [16]. However, genome-wide, no correlation has been observed between copy number and gene expression, even in chromosomal arms completely amplified like 5p or 3q. In cell lines having 5p amplified, only 22% of the investigated genes were upregulated. Similarly, invasive tumors or cell lines having amplified 3q showed even a lower proportion of upregulated genes [16]. The lack of correlation between copy number (CN) and gene expression [16], [31] suggests that some of the altered regions identified by SNP or mCGH arrays could be CN-altered discontinuously and not all genes within the regions are affected. However, the role of copy number in gene deregulation has not been studied in detail genome-wide and only particular regions have been investigated [16], [31], [33]. In this study, we investigated whether the CN alterations in cell lines, genome-wide on a gene-by-gene level, were associated with changes in gene expression. For that purpose, the CN alterations of the whole genome and the level of expression of over 20,000 genes were explored in 4 cell lines by using the 100 K SNP and Human Gene 1.0 ST microarrays from Affymetrix.

## Results

### Identification of genes potentially altered in copy number

**Figure 1:**
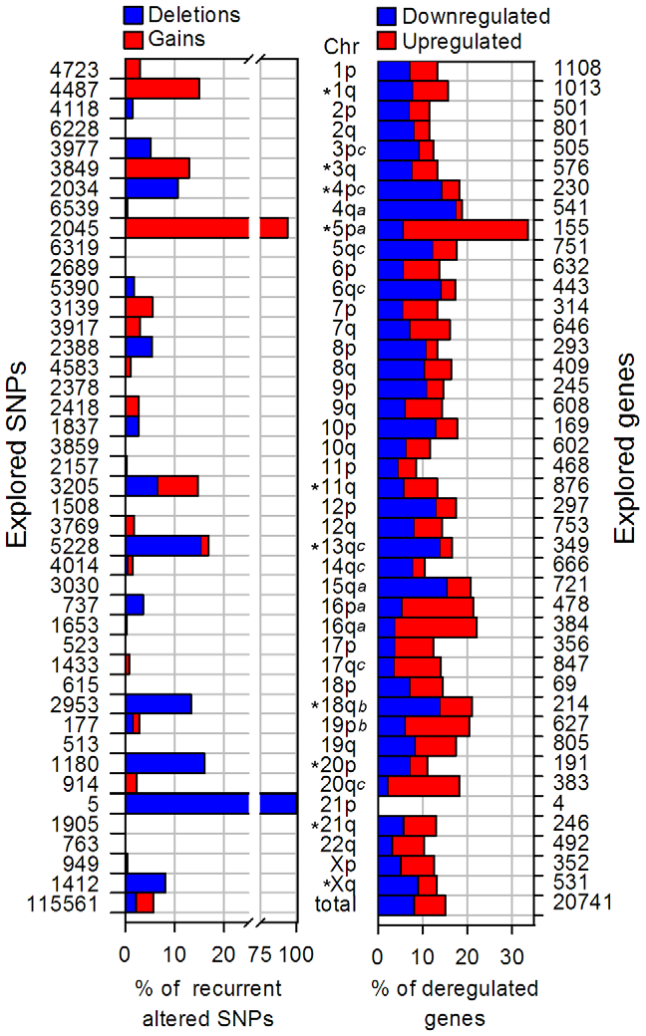
Comparison between copy number changes and gene expression by chromosomal arms. On the left side, the number of SNPs located in each chromosomal arm is indicated, which were explored by the 100 K microarray. On the right side, the number of genes located in each arm is indicated, which were explored for changes in gene expression by the ST1.0 expression microarray. Each bar represents the percentage of recurrent altered SNPs (left) or deregulated genes (right) common to the 4 cell lines. The chromosomal arms are indicated in the middle column. Arms labeled with asterisks had a mean number of CN-altered SNPs higher and statistically significant (p<0.05, chi-square test) compared with the whole genome means. Arms with a statistically significant deregulated gene enrichment were labeled with “a” (identified with both chi-square test and PAGE), “b” (identified only with chi-square test, p<0.05) or “c” (identified only with PAGE).

**Figure 2:**
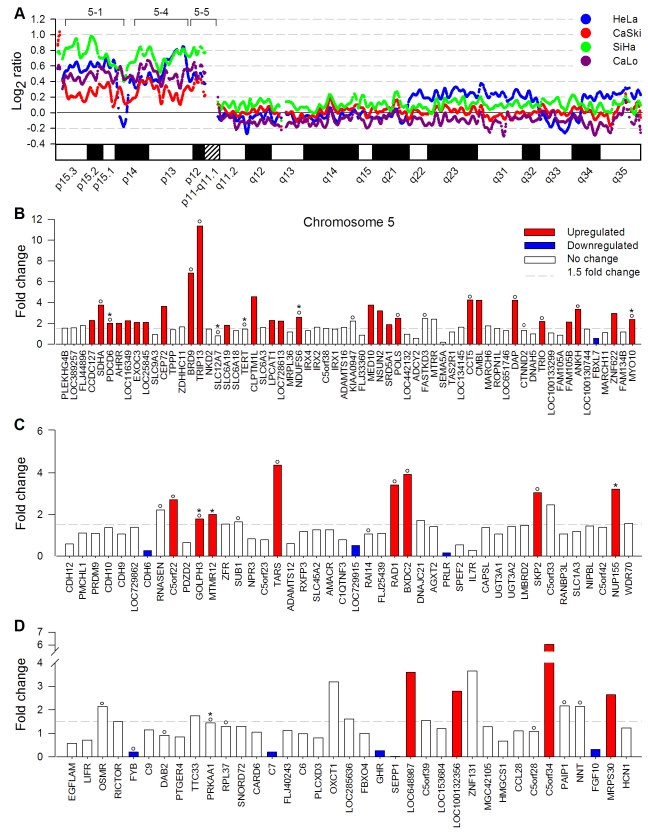
Genome amplification and deregulation of gene expression in Chr 5p. Panel A shows the copy number log_2_ ratio of SNPs investigated in Chr 5 by the 100 K SNP microarray in HeLa, CaSki, SiHa, and CaLo. Panels B to D show the fold change of gene expression of genes evaluated by the ST1.0 expression microarray located in MRR 5-1 (n = 64), MRR 5-4 (n = 44), and MRR 5-5 (n = 37) at 5p. The bars represent upregulated genes, downregulated genes, and genes without change in gene expression. The genes are ordered according to position in the genome. The SAM method was used for the analysis, using cut-off values of fold change of ≥1.5 or ≤0.66 for up- or downregulated genes and fold discovery rate (FDR) of 0%. Genes previously reported associated with cervical cancer are labeled with asterisks (IPA system) or circles (PubMed).

Each cell line had on average 49,167 copy number-altered SNPs (CN-AS; 42.6% of all SNPs evaluated), mostly gains (45.5%) and single deletions (48.5%). Amplifications (5.5%) and, particularly, double deletions (0.5%) were rare events. A total of 1,065 different CNAs was identified in the 4 cell lines investigated, 599 gains and 466 deletions. On average, cell lines had 273±32 (range, 240–317) CNAs and the overall CN-altered genome was about 19.2%. When the CNAs of the 4 cell lines were aligned (Figure S1), 108 minimal recurrent regions (MRRs) were identified (Table S1). They had a mean size of 787 kb (range, 3.4–16,755 kb) and the amount of DNA included in the whole set of MRRs corresponded to 2.4% of the genome. The recurrent SNPs that constituted the MRRs represented only 5.8% of all evaluated SNPs. Only 10 chromosomal arms had a greater and statistically significant rate (labeled with an asterisk in Figure 1). Three of them had only gained SNPs (1q, 3q, and 5p), 6 had only deleted SNPs (4p, 13q, 18q, 20p, 21p, and Xq), and 1 (11q) had both gained and deleted SNPs. It is notable that 94% of the 2,045 evaluated SNPs in 5p was amplified, suggesting that the whole 5p was duplicated (Figure 2A). The percentage of altered SNPs in the other arms was much lower than in 5p, except in 21p, which had 100% altered SNPs. However, there were only 5 evaluated SNPs in this arm (Figure 1). On the other hand, 45 out of 297 cytobands had a significant higher rate of recurrent altered SNPs than the whole genome, most of them are located in the chromosomes identified above (Table S2). Cytobands with the higher rates included 9 amplified (1q31, 5p12, 5p13, 5p14, 5p15, 3q24, 3q26, 7p11, 7q32) and 6 deleted (4p16, 11q23, 11q25, 13q12, 13q14 and 18q11).

Assuming that CNAs and MRRs were continuously altered regions, to identify genes with CN alterations, they were aligned with the total human genes according to their position in the genome (Figure S1). The number of altered genes per cell line ranged from 6,864 in CaLo to 17,829 in SiHa (average, 11,669 genes; Table 1). Interestingly, 14 MRRs lacked genes and the number of genes in the remaining MRRs (n = 94) ranged from 1 to 103. A total of 1,264 genes was located in the MRRs, 619 deleted, 626 gained, and 19 deleted in some cells and gained in others (Table S3).

**Table 1:** Influence of copy number alterations in gene deregulation in cervical cancer cell lines.

		Frequency of deregulated genes^a^							
		CN+			CN−			All	
Cell line	Genes^b^ CN+	n	EX+	%	n	EX+	%	EX+	%
CaLo	6,864	4,487	532	11.9	16,254	1,686	10.4	2,218	10.7
CaSki	7,706	4,912	728	14.8	15,829	2,197	13.9	2,925	14.1
HeLa	14,276	8,875	2,207	24.9	11,866	3,088	26	5,295	25.5
SiHa	17,829	11,120	1,125	10.1	9,621	944	9.8	2,069	10
Average	11,669	7,349	1,148	15.6	13,393	1,979	14.8	3,127	15
Recurrent genes^e^	1,264^c^	783	147	18.8	19,958	2,975	14.9	3,122^d^	15.1

a 20,741 genes were explored for changes in expression with HG-ST1.0 microarray. On average 7,349 of them were CN-altered (CN+) and 13,393 did not have copy number alterations (CN−).

b Potentially copy number altered genes according to data obtained with 100 k microarray. On average only 63% of those genes were explored for changes in gene expression (numbers in column n of CN+ subset).

c Genes who were copy number altered in the four cell lines.

d Genes who were found deregulated when all four cell lines, by triplicate, were compared together against the control sample (n = 10).

e In the genome, included genes that were found in MRRs, and in the transcriptome, include genes which were found deregulated uniformly in the four cell lines (described in d).

EX+ = Genes that were up- or down- regulated in cancer cell lines compared with the control sample.

### Gene expression analysis of 20,741 genes in CC cell lines

The amount of mRNA transcribed from 20,741 genes was compared between individual cell lines or all 4 cell lines together and 10 normal cervical epithelial controls. The raw data was standardized with the robust microchip average (RMA) algorithm of the FlexArray software, and genes with a different expression level were identified with the “Significance Analysis of Microarrays” (SAM) method using cut-off values of fold change of ≥1.5 and a fold discovery rate (FDR) of 0% (see Materials and Methods). The mean number of genes expressed differentially between cancer cells and the control group was 3,127 and ranged from 2,069 (10%) in SiHa to 5,295 (25.5%) in HeLa (see Table 1). When experiments of the 4 cell lines were taken together as a group, 3,122 genes (15.1%) were found expressed differently compared with that of the control group, 1,434 upregulated and 1,688 downregulated (Table S4).

The frequency of deregulated genes was calculated in each chromosomal arm and cytoband. Only 7 chromosomal arms (4q, 5p, 15q, 16p, 16q, 18q and 19p) showed a greater and statistically significant (p<0.05, chi-square) percentage of deregulated genes compared with that of the whole set (15.1%; Figure 1). Arm 5p had the greatest percentage (33.5%) of deregulated genes followed by 16q (22.1%), 16p (21.3%), 18q (21%), 15q (20.8%), 19p (20.4%) and 4q (18.9%). In 5p, 16p,16q and 19p the upregulated genes predominated, whereas in 4q, 15q and 18q the downregulated genes predominated (Figure 1). Only 10 out of 297 cytobands had also a greater and statistically significant rate, most of them located in these chromosomes (Table S2). Cytobands with the higher rates were 19p12 (61.8%), 15q11 (52.5%) and 5p15 (45.1%). Most of these Chr and cytobands were confirmed with the PAGE analysis (Figure 1 and Table S2), which consider in the calculations, besides the number of deregulated genes, the average value of fold change (FC; see material and methods). However, 8 Chr and 29 cytobands, not uncovered with the chi square test, were also detected enriched of deregulated genes with this method (labeled with a superscript “c” in Figure 1, Table S2), indicating that unlike the percentage, the mean values of FC were significantly different compared with the global average.

**Figure 3:**
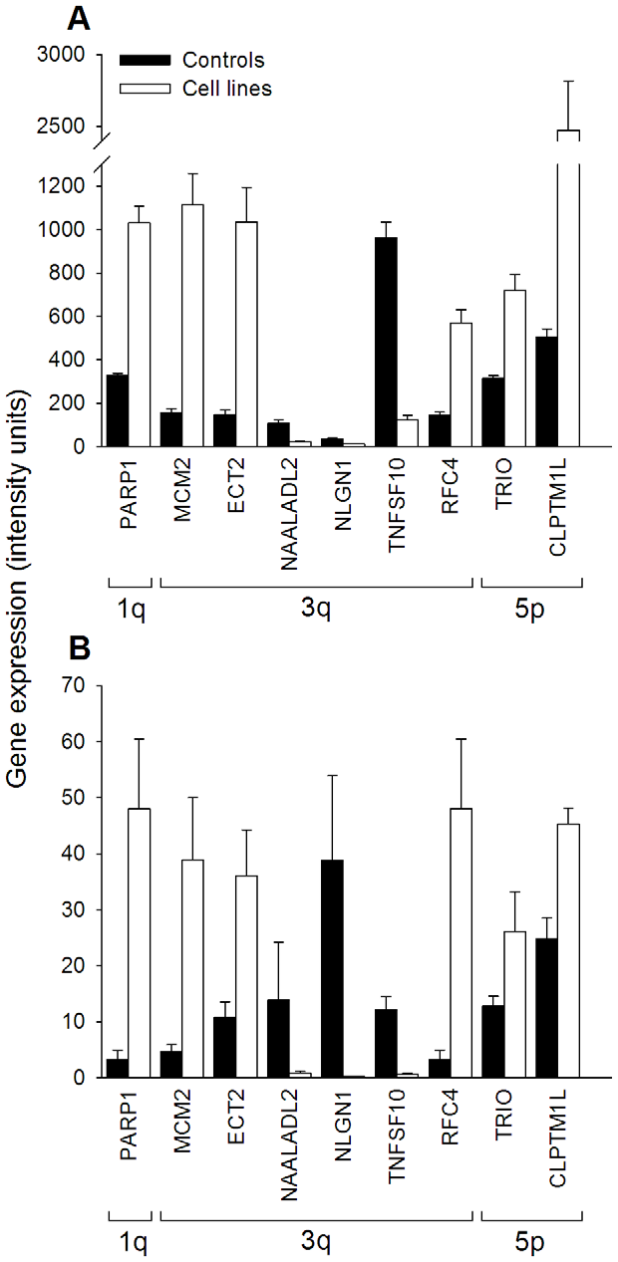
Comparison of gene expression of *PARP1*, *MCM2*, *ECT2*, *NAALADL2*, *NLGN1*, *TNSF10*, *RFC4*, *TRIO* and *CLPTM1L* between cell lines and controls. Panel A shows the experiments of microarrays and panel B the qRT-PCR experiments. Panels show the mean ± standard error of expression intensity of 9 CN-altered genes located at 1q (*PARP1*), 3q (*MCM2*, *ECT2*, *NAALADL2*, *NLGN1*, *TNSF10* and *RFC4*) and 5p (*TRIO and CLPTM1L*). For both methods intensities are expressed in relative units (see Materials and Methods).

A high statistically significant positive correlation (p<0.01) was found between the values of qRT-PCR and micro-arrays in all 23 genes evaluated with both methodologies. The correlation coefficients ranged from 0.61 to 1.0 and the average was 0.82. Figure 3 shows the mean intensity of mRNA of 9 genes located at 1q (PARP1), 3q (MCM2, ECT2, *NAALDL2*, *NLGN1*, TNFSF10 and *RFC4*) and 5p (*TRIO, CLPTM1L*), which were evaluated with qRT-PCR and microarrays. These experiments suggested that the whole data set of HG1.0ST microarrays was reliable.

### Correlation of copy-number alterations with gene expression

#### Analysis of gene expression in the whole set of CNAs and MRRs

Only 63% of CN-altered genes could be evaluated for changes in gene expression with the microchip HG1.0ST. On the other hand, from the 20,741 genes investigated for changes in expression, on average 35.4% of them were identified with potential alterations in the copy number in the cell lines (Table 1). The proportion of deregulated genes was slightly higher in the group of genes with CN alterations than in the group of genes without CN alterations (15.6% vs. 14.8%; p>0.05, chi-square). A higher difference was found in the group of recurrent altered genes (18.8% vs. 14.9%; p = 0.0035, chi-square). These small differences could suggest either that most genes identified with potential alterations in the copy number are not actually deleted or gained or that they are altered in copy number without changes in gene expression. In the first case, the CNAs might have been CN-altered discontinuously. In the second, the CNAs were presumably CN-altered continuously, but gene expression might have been modulated by other factors. Interestingly, on average, 69.1% of potentially CN-altered genes in each cell line did not have altered SNPs; rather, they were located between 2 altered SNPs within the CNAs (Figure S1). The rest (30.9%) had from 1 to 282 altered SNPs (mean, 6±10). However, it was expected that genes located in a fully altered region would be deregulated similarly, regardless of number of SNPs.

**Figure 4:**
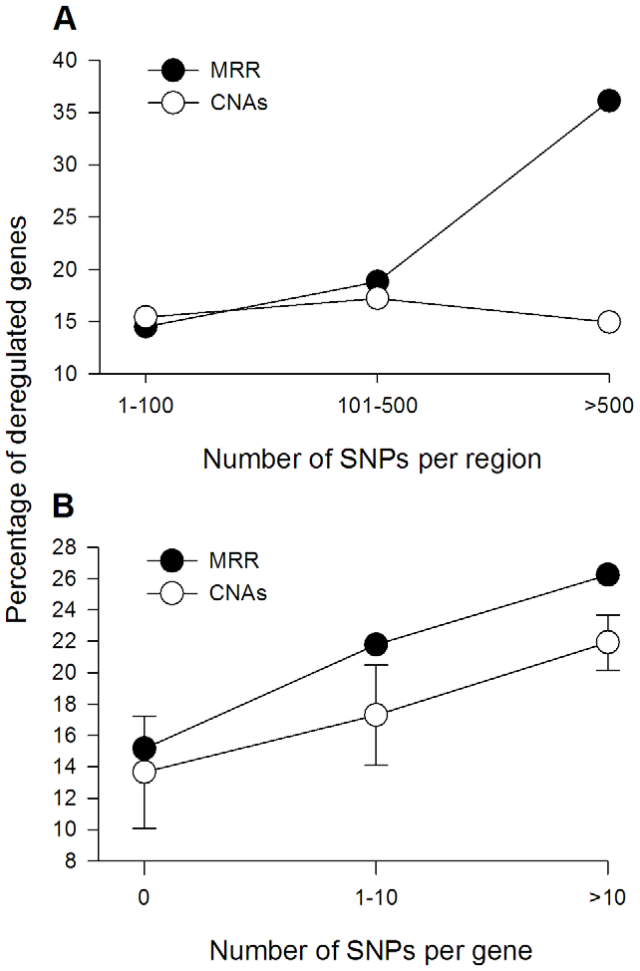
Trend of deregulated genes as CN-altered SNPs increased by gene or region. The figure shows the percentage trends of deregulated genes as the number of CN-altered SNPs per region (panel A) or gene (panel B) increased. MRR includes the genes harbored by the minimal recurrent regions common to the 4 cell lines. The linear association between the variables in all but one plot (CNAs, panel A) was statistically significant, p<0.01, Mantel–Haenszel linear-by-linear association chi-squared test.

One indirect way to test this hypothesis globally was to investigate whether the percentage of deregulated genes rises as the number of SNPs/gene or region increases. In the whole set of CNAs, the rate of deregulated genes did not increase as the number of SNPs per CNA increased, rather it remains uniform around 15.6% (Figure 4A). By contrast, in the MRRs, the trend increased from 14.5% in genes located in MRRs with 1–100 SNPs to 36.1% in genes located in MRRs with more than 500 SNPs (p<0.001, Mantel–Haenszel linear-by-linear association chi-squared test; Figure 4A). The numbers also increased with the density of altered SNPs, from 16.5% in genes located in MRRs with more than 20 kb/SNP to 23.2% in genes located in MRRs with less than 20 kb/SNP (p = 0.03, Pearson chi-square; data not shown). This data suggest that MRRs with the higher number of SNPs are more likely to be completely CN altered. However, the fact that the percentage of deregulated genes increased linearly as the number of altered SNPs per gene increased (p<0.01, Mantel–Haenszel linear-by-linear association chi-squared test; Figure 4B), suggests that many of those regions were CN-altered discontinuously.

**Figure 5:**
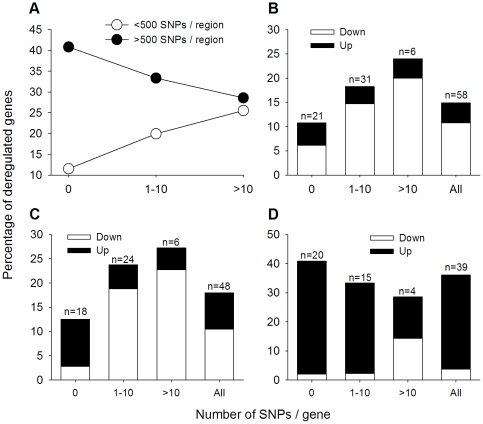
Trend of down- and upregulated genes as CN-altered SNPs/gene increased in deleted and amplified MRRs. In panel A, the percentage trend of deregulated genes is compared among the genes located in MRRs having 1–100, 101–500, and >500 SNPs. The trends of up- and downregulated genes are shown in panels B (47 deleted MRRs, <500 SNPs), C (51 amplified MRRs, <500 SNPs), and D (2 amplified MRRs, >500 SNPs). The total number of genes studied for expression and included in the analysis of panels B, C, and D was 390, 267, and 108, respectively. The numbers above the bars indicate the number of deregulated genes.

A stratified analysis was performed to clarify the precise relationship among these variables. In the pool of CNAs, the percentage trend of deregulated genes increased with the number of SNPs/gene, either they were located in CNAs having less or more than 500 SNPs or low or high density of SNPs (data not shown). This data suggests most CNAs are not CN-altered continuously. In the set of recurrent altered genes, the trend of deregulated genes differed if they were located in MRRs with less or more than 500 SNPs. In the former group, the trend was similar to that observed in the whole set of CNAs (p<0.001, Mantel–Haenszel linear-by-linear association chi-squared test; Figure 5A), suggesting they were also CN-altered discontinuously. It is notable that in the discontinuous CNAs (data not shown) or MRRs, the ascendant trend is due to downregulated genes, either they were deleted (for MRRs, p = <0.01, Spearman's correlation; Figure 5B) or amplified (for MRRs, p<0.01, Spearman's correlation; Figure 5C). In the case of the 2 amplified MRRs having more than 500 SNPs, although the trend of de-regulated genes declined slightly from 40.8% to 28.6% with the number of SNPs/gene (Figures 5A), it was not statistically significant (p = 0.333, Mantel–Haenszel linear-by-linear association chi-squared test). It is notable that this subset of MRRs showed the greatest percentage of de-regulated genes (36.1%, 39 out of 108; Figure 5D), with over 89.7% upregulated (35 out of 39; Figure 5D). This data suggest these MRRs are more likely to be completely CN altered. Interestingly, these 2 MRRs are located at 5p, the arm already demonstrated as fully amplified. One (MRR 5-1) is located in cytoband 5p15 and the other (MRR 5-4) is located in cytoband 5p14 (Table S1).

#### Gene expression analysis by individual MRR

Another way to investigate the actual copy-number status of MRRs is by comparing the percentage of deregulated genes in each MRR with that of the whole set of MMRs. In principle, it is expected that where the MRR is actually deleted or gained, most of the genes located within that MRR should change expression in the same way as the CN alteration. Only 61.9% (783 of 1264) of genes located in 85 MRRs was investigated for changes in expression. The percentage of total deregulated genes was 18.8% (147 of 783) and only 32 MRRs had a higher than this percentage. However, only in 4 of them, 2 gained (MRR 3-13 and 5-1; Table S1) and 2 deleted (MRR 4-4 and 13-2; Table S1), the difference against the whole set (18.8%) was statistically significant. Of particular note is MRR 5-1, because 43.8% of the investigated genes (28 out of 64) were deregulated and the p value was very low (4.5×10^−6^; chi-square). Of these, 27 genes were upregulated and only 1 (*FBXL7*) was downregulated (Figure 2B). As expected, the percentage of overexpressed genes was similar in the subgroups of genes with (50%) or without (38.9%) altered SNPs (p>0.05, chi-square; data not shown). The high percentage of upregulated genes in both subgroups of genes strongly suggests that this MRR is fully duplicated. The other 3 regions (MMRs 3-13, 4-4 and 13-2) had a higher percentage of deregulated genes (57.1%, 75% and 46.2, respectively), but they only had limited genes evaluated for expression and the p values were just below 0.05 (Table S1). It is important to note that if all genes located in MRRs were explored and deregulated genes were found in the same proportions, the difference of these 3 MRRs from the mean would be stronger, and 2 additional MRRs could be identified (MRRs 1-4 and 19-2; Table S1). Interestingly, the MRR 3-13 is located in 3q26, a cytoband frequently amplified in CC. The other potentially gained MRR containing more than 500 SNPs (MRR 5-4), identified in the analysis showed above, did not have a percentage of deregulated genes much higher than the mean to be statistically significant (p>0.05; chi-square test), because only 25% (n = 11) of the 44 genes investigated for expression were deregulated.

#### Gene expression analysis by chromosome arms and cytobands

**Figure 6:**
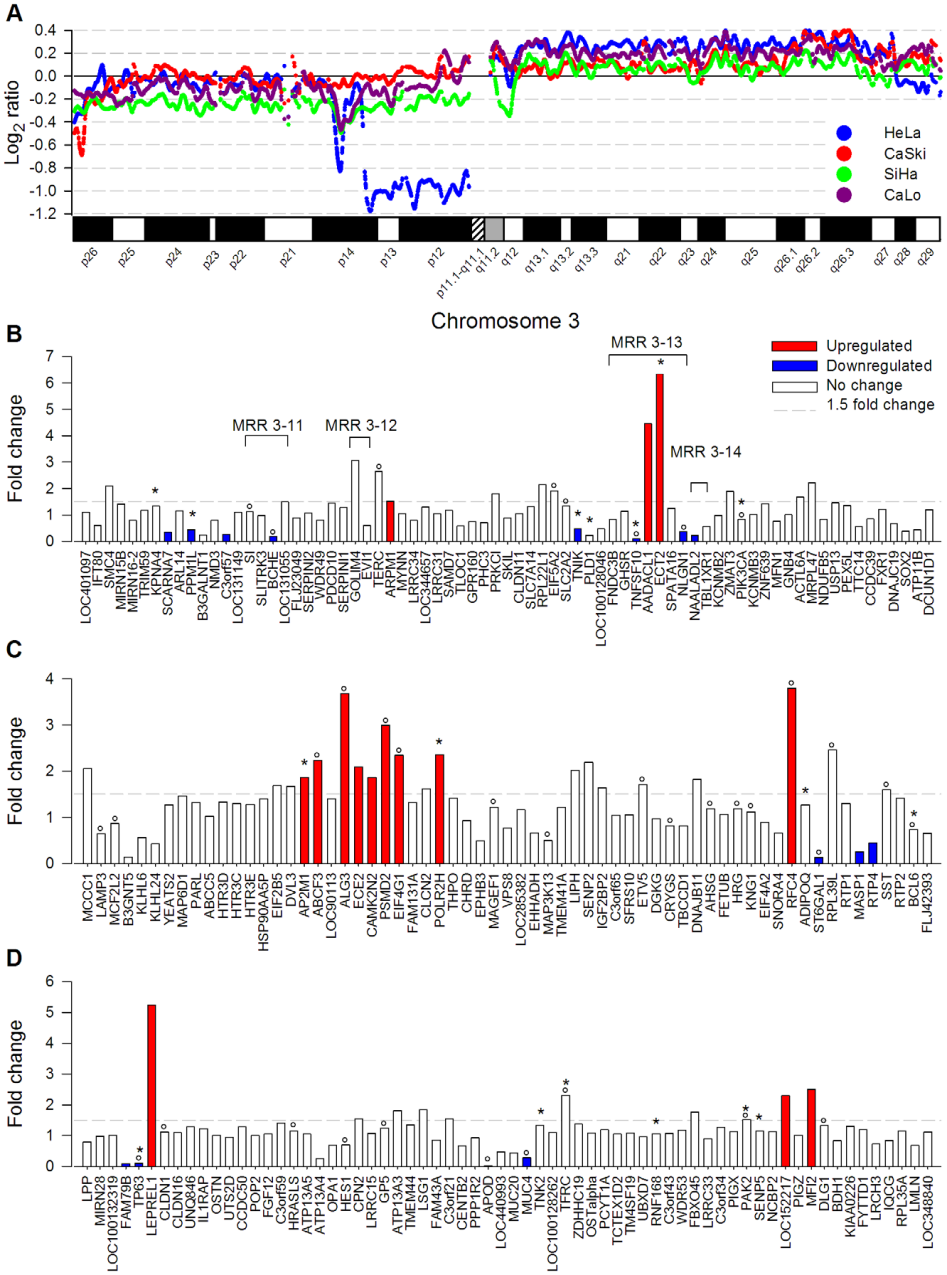
Genome amplification and deregulation of gene expression in 3q. Panel A shows the copy number log_2_ ratio of SNPs investigated in Chr 3 by the 100 K SNP microarray in HeLa, CaSki, SiHa, and CaLo. Panels B to D show the fold change of gene expression of genes evaluated by the ST1.0 expression microarray located at 3q26 (n = 73), 3q27 (n = 63), and 3q28–29 (n = 66). The genes are ordered according to the position in the genome. See the legend of Figure 2 for further information.

The correlation of CN and gene expression analyzed by chromosomal arms and cytobands was very poor. Only 5p showed a clear correlation between CN and gene expression (Figure 1), because the high percentage of recurrent gained SNPs (94%) correlated with a high proportion of upregulated genes (27.7%). The correlation was specially higher in 5p15 and 5p12 where the rate of upregulated genes increased up to 45.1% (Table S2). To a lesser extent, higher percentages of deleted SNPs were correlated with the enrichment of downregulated genes in 4p, 13q and 18q (Figure 1). The other 6 arms and 37 of the 43 remaining cytobands, identified with a high percentage of CN-AS, did not show any gene enrichment compared with the whole genome, including 3q (13.4%) and 3q26 (15.1%), which had exclusively gained SNPs but downregulated genes predominated (7.8% in 3q, Figure 1; and 11% in 3q26, Table S2). When 3q was analyzed separately in each cell line, a high proportion of gained SNPs was found in CaLo (93.5%) and HeLa (87.2%; Figure 6). However, the proportion of deregulated genes increased only about 2 fold in HeLa (23.4%) but not in CaLo (12.7%) compared with that in CaSki (13.9%) and SiHa (9.4%). On the other hand, 4q, 5q, 6q, 14q, 15q, 16q, 16p, 17q, 19p and 20q, which also showed an enrichment of deregulated genes, had none or a very low percentage of CN-AS (Figure 1). This is also the case for 31 of the 39 cytobands that showed a significant enrichment of deregulated genes. It is especially notorious in 15q11 and 19p12, which did not have recurrent altered SNPs, but showed downregulated more than 50% of the explored genes (Table S2).

#### Analysis of 5p, 3q and 1q

Although the full 5p arm seemed to be amplified (Figure 2A), it is worth noting that around 2/3 of genes located in this arm were not deregulated, and from those de-regulated genes, 9 were found downregulated (Figure 2B–D). Furthermore, the proportion of deregulated genes was not evenly distributed along 5p, because it was much higher in MRR 5-1 (5p15; 43.8%; Figure 2B) than in MRR 5-4 (5p14.3-5p13.2; 25%; Figure 2C) and MRR 5-5 (5p13.1-5p12; 24.3%; Figure 2D). Downregulated genes were almost absent in MRR 5-1 but increased in MRRs 5-4 and 5-5. It is notable that 48.8% of deregulated genes in 5p, especially in MRR 5-1, were distributed in clusters of 2 or more contiguous deregulated genes (Figure 2B). This distribution was statistically significant from a random distribution (p<1×10^−8^, chi square test), suggesting that besides the segment amplification, the location of genes within the same region of chromatin, perhaps at a loop level, may influence gene expression. Several genes previously reported and supposed to be involved in cervical carcinogenesis, like *BRD9*, *POLS*, *SDHA*, and *TRIO*, were also identified upregulated and located in MRR 5-1 (Figure 2B).

Figure 6 shows the intensity (log_2_ ratio) of SNPs investigated in Chr 3 (Figure 6A) and the expression fold change of genes evaluated at 3q26–29 (Figure 6B–D), where there are genes frequently identified or associated with CC. Only 3q26 had MRRs (MRRs 3-11, 3-12, 3-13, and 3-14; Figure 6B). A very low percentage of deregulated genes per cytoband is shown (∼13%), particularly in 3q28/3q29. However, 31.9% of deregulated genes, similarly to those investigated in 5p, were located together in groups of 2 or more genes, intercalated with several genes with no changes in gene expression. In the case of 3q26 (Figure 6B), there is one cluster, aligned with MRRs 3-13 and 3-14, including 3 downregulated (*TNFSF10*, *NLGN1*, and *NAALADL2*) and 2 upregulated genes (*AADACL1* and *ECT2*). On the other hand, there are 1 MRR (3-12) that had genes with no changes in gene expression. In 3q27, there is a cluster with 5 overexpressed genes (*ALG3*, *ECE2*, *CAMK2N2*, *PSMD2*, and *EIF4G1*). It is noteworthy that genes like *TERC*, *PIK3CA* (3q26), and *LAMP3* (3q27) were neither CN altered recurrently nor upregulated (Figure 6B and C).

**Figure 7:**
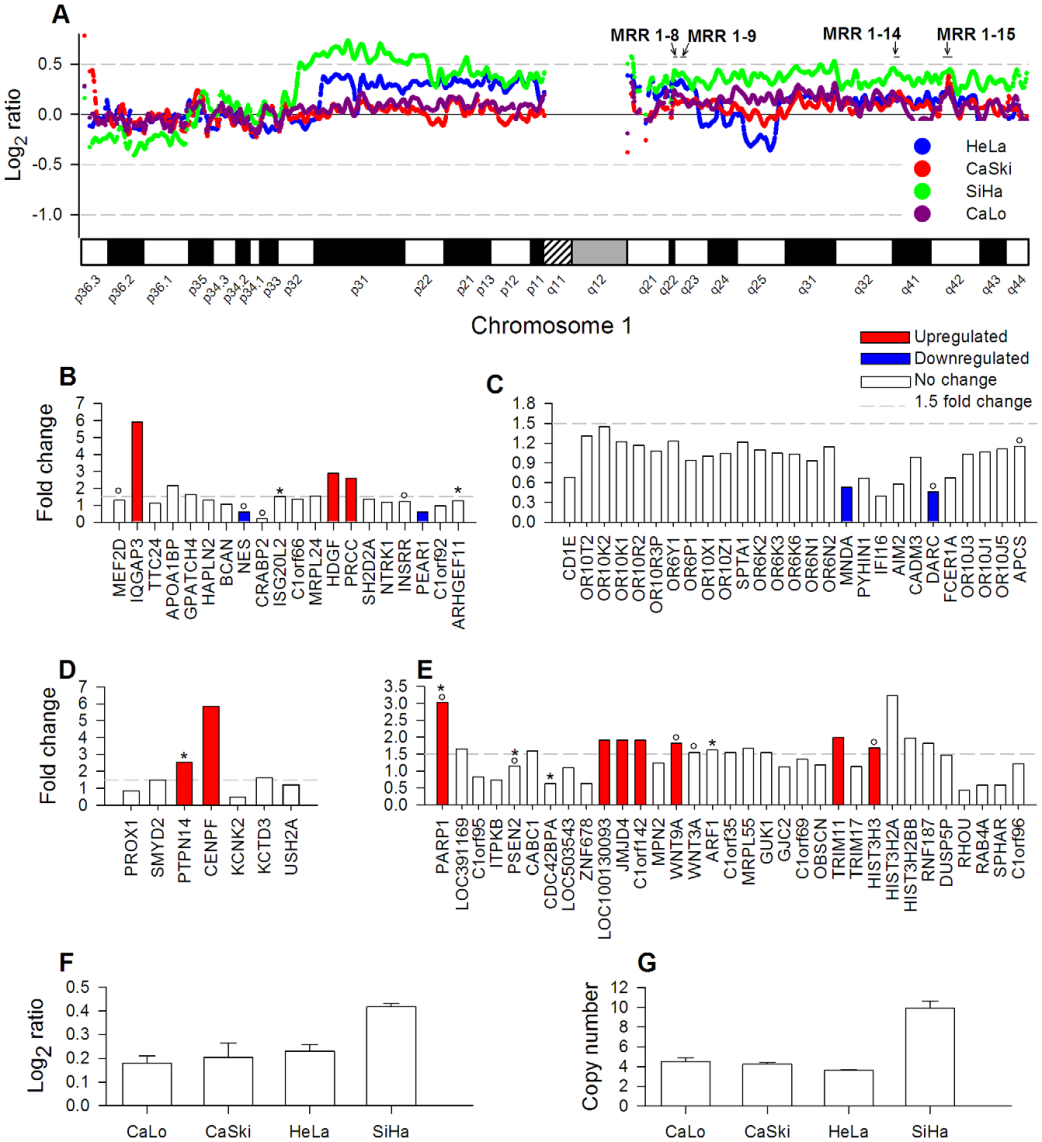
Genome amplification and deregulation of gene expression in Chr 1q. Panel A shows the copy number log2 ratio of SNPs investigated in Chr 1 by the 100 K SNP microarray in HeLa, CaSki, SiHa, and CaLo. Panels B to D show the fold change of gene expression of genes evaluated by the ST1.0 expression microarray located at MRRs 1-8, 1-9, 1-14 and 1-15. The genes are ordered according to position in the genome. For panel F, the mean ± S.D. of the log2 ratio signal of 110 SNPs located in the MRR-15 was plotted. In panel G is shown the copy number of PARP1 gene calculated by qPCR in triplicate experiments. See the legend of Figure 2 for further information.

Figure 7 shows the signal intensity (log_2_ ratio) of SNPs investigated in Chr 1 (Figure 7A) and MRR 1-15 (Figure 7F), the expression fold change of genes evaluated at 4 MRRs (1-8, 1-9, 1-14 and 1-15; Figure 7B–E) and the copy number of PARP1 gene evaluated by qPCR (7G). Similarly to 3q, the percentage of deregulated genes per cytoband in 1q was very low (mean = 13%) and only 1q21 had a higher rate than the whole genome (22.2%, Table S2). Even in the MRRs, only an average of 17% of genes was de-regulated (calculated from Table S1) and the small difference (2.5%), compared with the whole 1q (14.5%; Figure 1), was not statistically significant. In figures 7B–E are shown the MRRs (1-8, 1-9, 1-14 and 1-15) which had the greater number of genes explored for changes in expression (Table S1). In MRR 1-9 (Figure 7C), it is notorious that none of the 27 genes explored were upregulated, instead two of them were downregulated (MNDA and DARC). In contrast, in MRR 1-15 (Figure 7E), 7 out of 33 (21.2%) explored genes were upregulated, including PARP1. Similarly to 5p and 3q, the deregulated genes were often (37.9%) located together in groups of 2 or more genes intercalated with several genes with no changes in gene expression. This is clearly seen in MRRs 1-8 (Figure 7B), 1-14 (Figure 7D) and 1-15 (Figure 7E). The amplification of PARP1 gene was validated in the four cell lines by qPCR with a TaqMan assay (Figures 7G). Interestingly, the number of copies correlated with the mean intensity (log2 ratio) of the 110 SNPs located in the MRR 1-15 explored with the 100 K microarray (Figure 7F). Whereas CaLo, CaSki and HeLa had about 4 copies of PARP1 gene and a log2 ratio around 0.2, SiHa had 10 copies and a log2 ratio of 0.4.

### Fluorescent in situ hybridization (FISH)

**Figure 8:**
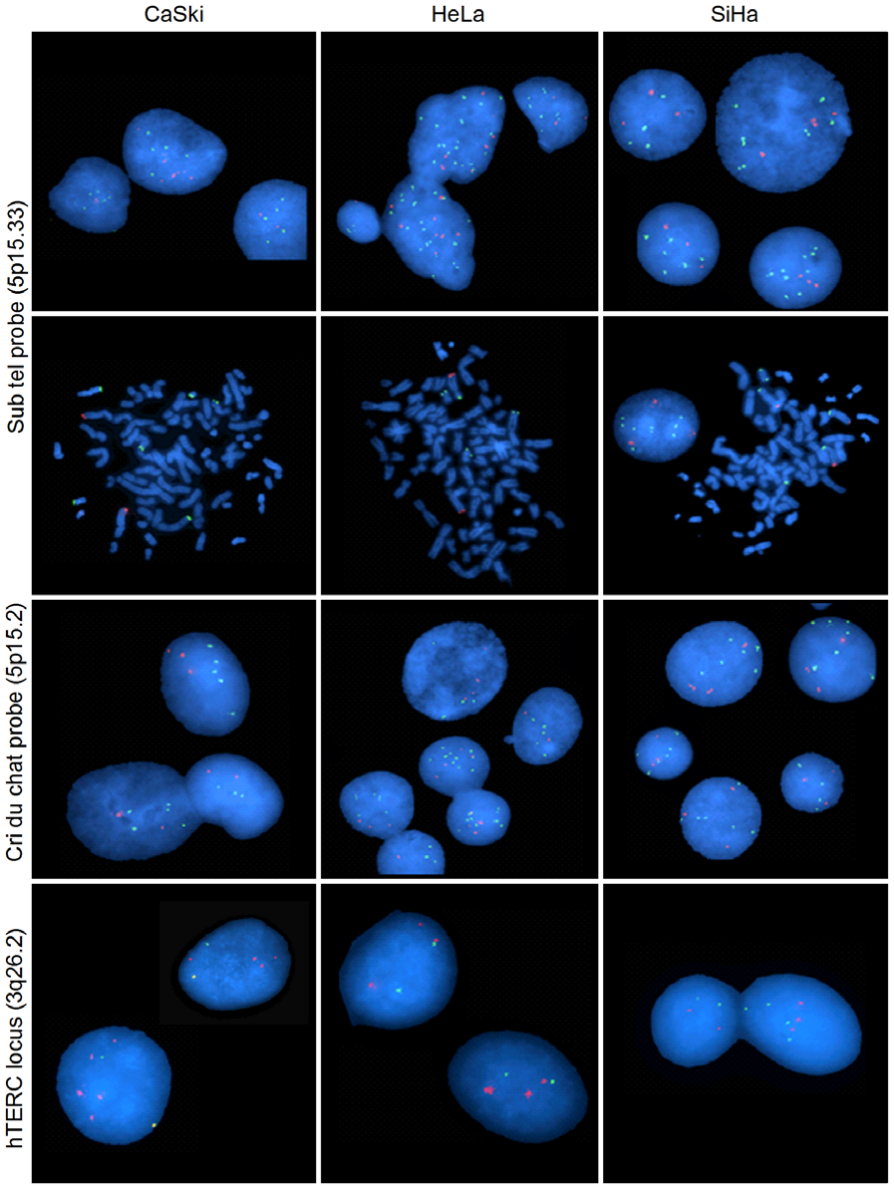
Copy number analysis of cytobands 5p15 and 3q26 with fluorescence in situ hybridization (FISH). Representative experiments of FISH analysis of cytobands 5p15.33, 5p15.2, and 3q26 in 3 cell lines (CaLo, CaSki, and HeLa) are shown. Two sets of probes were used for the analysis of 5p15 and one for the analysis of 3q26 (see Materials and Methods). The sets for 5p15 included a target probe (green signals) and a control probe (red signals) located at 5q. The set for 3q26 included a target probe (green signals) and a control probe located at the centromere. Nuclei in interphase (first, third, and fourth rows) and Chr in metaphase (second row) were counterstained with DAPI.

The copy number of the 5p15 region, where MRR 5-1 is located, was investigated in 3 cell lines (CaSki, SiHa, and HeLa) with FISH using a sub-telomeric probe at 5p15.33 and a locus-specific probe at 5p15.2 (green signals in Figure 8; see Materials and Methods). The copy number was estimated by comparing the signal of the target probes with that of internal control probes located at 5q35.3 and 5q31 (red signals in Figure 8). The 2 probes showed amplification at the 5p15 region but the signal ratio of the probe located at 5p15.33 was higher (Figure 8). The signal ratio was 2.3 (range, 2.3–2.4) at 5p15.33 and 2 (range, 1.8–2.2) at 5p15.2. The difference in the fold change between the 2 probes was noteworthy in CaSki (2.4 vs. 1.8) but in full concordance with the microarray data, because 5p15.3 was found highly amplified, whereas the rest of 5p15 was found less amplified (Figure 2A). The Chr spreads showed recognized genomic instability in all cell lines, complex aberrations of Chr 5 including 5p isochromosomes and 5p centric and acentric fragments, and double minutes (Figure 8). In agreement with the microarray data, 3q26.2 was confirmed amplified in CaSki and HeLa but not in SiHa (Figure 8). However, the signal ratios in HeLa (1.2) and CaSki (1.7) were lower than those observed in 5p15.

### Classification of genes that expressed differentially or had potential alterations in copy number

The DAVID functional annotation tool (http://david.abcc.ncifcrf.gov) was used to identify the biological processes where the 3,122 genes differentially expressed, the CN-altered genes found in each cell line and the 147 deregulated and recurrent CN-altered genes are involved. Compared with the human genome database, the 3 physiological processes more enriched in the former set of genes and with the lowest p values were the cell cycle (169 genes, fold change [FC] = 1.9, p = 6×10^−18^), cell adhesion (178 genes, FC = 1.6, p = 2×10^−11^) and DNA metabolic processes (121 genes, FC = 1.5, p = 1.2×10^−6^). For the set of recurrent CN-altered genes with deregulated expression (147), the 3 most enriched physiological processes were phosphorus metabolic process (20 genes, FC = 2.7, p = 9×10^−5^), positive regulation of signal transduction (10 genes, FC = 4.5, p = 3.5×10^−4^), and the regulation of cell communication (19genes, FC = 2.4, p = 6×10^−4^). Interestingly, in the subset of upregulated genes (n = 28), belonging to the duplicated MRR 5-1, apoptosis was the only enriched process (FC = 5.2; p = 0.03). This was accounted for by 5 genes involved in the apoptotic process including CLPTM1-like (*CLPTM1L*), aryl-hydrocarbon receptor repressor (*AHRR*), programmed cell death 6 (*PDCD6*), death-associated protein (*DAP*) and triple functional domain (*TRIO*) genes. On the other hand, the analysis of the set of genes located in the potential CNAs did not show an enrichment of cancer-related process (data not shown). These data also support the hypothesis that most potentially CN-altered genes are not actually altered in the number of copies or deregulated.

**Figure 9:**
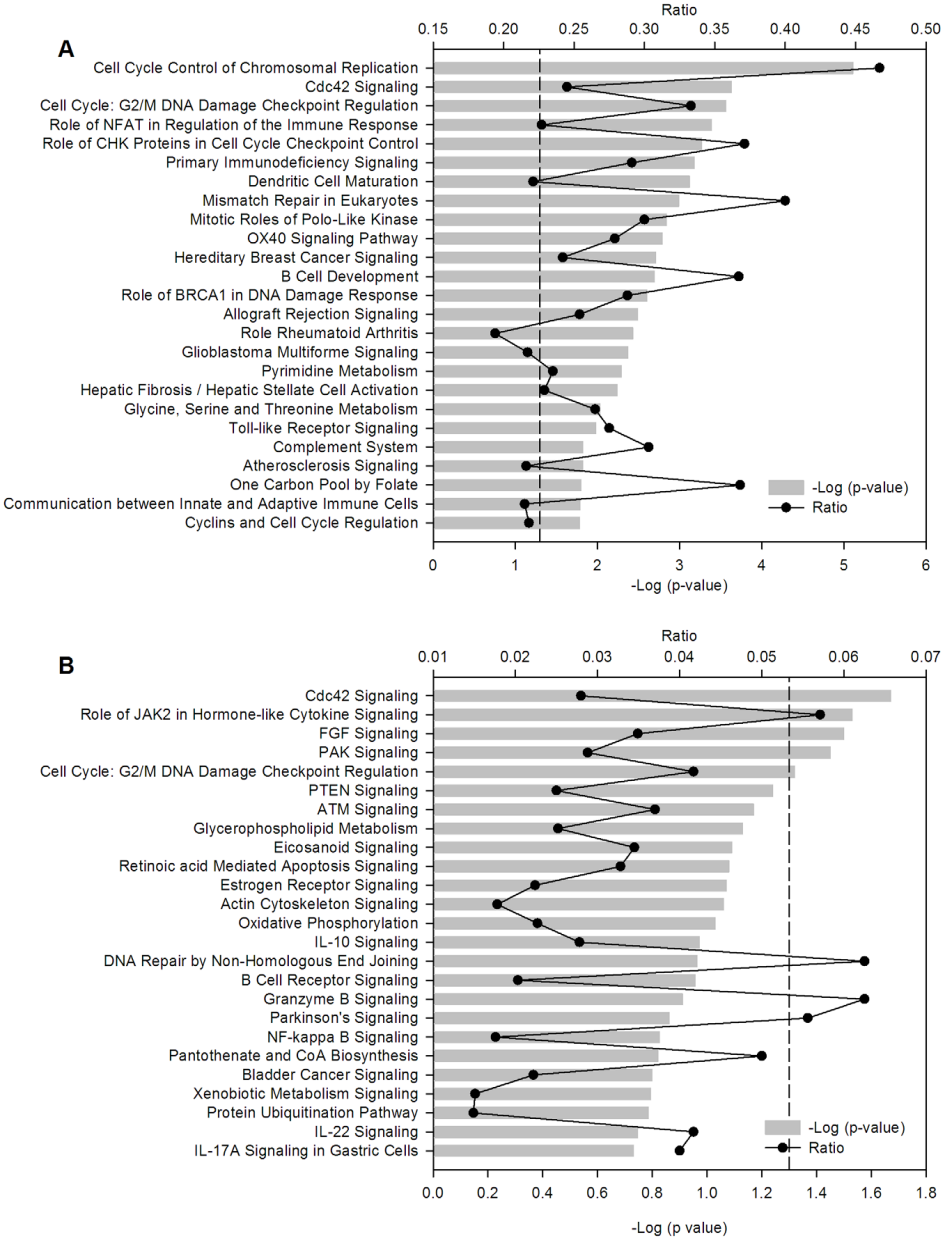
Canonical pathways where deregulated genes are involved. Top 25 canonical pathways identified in the set of 3,122 genes deregulated in the four cell lines (A) and in the subset of 147 deregulated and recurrent CN-altered genes (B). The canonical pathways were identified with the Ingenuity Pathway Analysis (IPA) system. The −log (p-value), gray bars, and the ratio, black dots, were calculated comparing the number of genes of the pathways present in the datasets versus the human database. The p-value was calculated with the chi square or Fisher exact tests as appropriate and the values of −log (p-value)>1.3 (dash line) correspond to p<0.05.

The data were also analyzed with the IPA Ingenuity system and the findings were very similar to those obtained with DAVID, at least for the larger groups. In agreement with the DAVID analysis, the top canonical pathways are from the cell cycle, followed by pathways of the immune system, in the group of 3,122 genes (Figure 9A) and from cell signaling and cell cycle in the group of 147 genes (Figure 9B).

## Discussion

In this study, we found a poor correlation between CN alterations and changes in gene expression. Only a small percentage of genes located in CNAs was de-regulated (15.6%), which was a minor difference (0.8%) from that of the subset of genes with non-CN alterations (14.8%). In the subset of CN-altered and deregulated genes, the way of deregulation was not necessarily the same as the CN alteration, *i.e.*, amplified genes were not always upregulated, instead they were often downregulated, and some deleted genes were found to be upregulated. This analysis was essentially based on the comparison of copy-number alterations and global gene expression investigated through genomic technologies and the characterization of certain regions by FISH and qPCR. The healthy normal epithelium of the cervix might not be the best control to measure the level of gene expression in cell lines. However, it is difficult to select an appropriate control, because these cell lines have been maintained in culture for many years. Although they do not fully represent the complexity of a tumor, they usually retain their genetic properties [34]–[36]. The use of primary cultures of normal cervical epithelium may be a better control, but there are not any available commercially. On the other hand, similar global results have been observed between cervical carcinomas and the cell lines reported in this study, when the same set of healthy normal controls were used (data not shown).

The results of this paper suggest that most of the CNAs in the evaluated cell lines, identified formerly with the 100 K microarray as succession of altered SNPs, are not continuously altered regions that include the full DNA segment defined by the altered SNPs. Rather, they appear to be composed of small or partial deletions or gains, where the altered SNPs are located, alternating with long stretches of normal DNA. On the other hand, the MRRs are more likely to be completely CN altered, because the proportion of deregulated genes was increased up to 3 fold, particularly in those having a high density or more than 500 SNPs. In those MRRs, the proportion of deregulated genes did not change with the number of SNPs per gene, further supporting they are entirely CN altered. In fact, the 2 MRRs having more than 500 SNPs (5-1 and 5-4), located at 5p, were confirmed as being completely amplified by FISH. In contrast, the finding that in MRRs composed of less than 500 SNPs, the percentage of deregulated genes increased with the number of SNPs/gene, strongly suggests those regions are CN affected in a discontinuous manner. However, it cannot be ruled out that some MRRs having less than 500 SNPs are completely CN altered, such as MRR 5-5 located at 5p, which had 346 SNPs and was confirmed fully amplified by FISH.

In previous studies, Chr 5p has often been found to be amplified in CC and cell lines derived from them, and many genes located in the region have been involved in the tumorigenic process [16], [20], [22], [30], [37]. Nevertheless, a rather poor correlation has been found between amplification and gene expression in this region, both in cell lines (22%) [16] and invasive tumors (18.9%) [22]. Although in the present study, a higher percentage of genes was deregulated (33.5%), a large proportion of the investigated genes was not deregulated. Furthermore, not all the deregulated genes were upregulated, 9 of them were downregulated. Because the entire 5p arm was demonstrated to be amplified by FISH (Figure 8), it is clear that not all amplified genes are upregulated, instead, some of them might be repressed, possibly by epigenetic mechanisms. The clustering of deregulated genes suggests that, besides the amplification of the segment, the location of genes within the same region of chromatin, perhaps at a loop level, may influence gene expression [38], [39].

The upregulated genes at MRR 5-1 may have a role in the carcinogenic process. They include *BRD9* and *POLS*, which participate in DNA repair and cell-cycle regulation, *SDHA* involved in mitochondrial oxidative phosphorylation [22], and *TRIO*, which promotes the exchange of GDP by GTP and could play a role in coordinating cell-matrix and cytoskeletal rearrangements necessary for cell migration and cell growth. In fact, *TRIO* has been associated with progression of bladder cancer [40] and soft tissue sarcomas. In the latter, a clear correlation has been demonstrated between amplification and gene upregulation [24]. Another upregulated gene located in this region was *CEP72*, which regulates the localization of key centrosome proteins involved in spindle formation [41]. This gene has been found frequently amplified in non-small-cell lung cancers [42]. *TERT*, which encodes the catalytic subunit of the telomerase complex hTERT and have been found to be amplified or upregulated in more than 90% of squamous cell cervical carcinomas and 40% of CIN III lesions [43], had a fold change of 1.45, just below the selected cut-off (Figure 2). The concordance between the upregulated genes found at 5p, in this and previous reports, was close to 60% [16], [22]. Most of the remaining genes that were shown to be upregulated in other studies had a fold change higher than 1.5 in this study but did not pass the delta score (Figure 2B–D; see Materials and Methods). However, additional genes linked to cancer processes were found deregulated in this study including *AHRR*, *C7*, *CLPTM1L*, and *MRPS30* involved in apoptosis, *CDH6* in cell adhesion and *CEP72* in the cell cycle.

In contrast with Chr 5, which shows clearly amplified 5p and unaltered 5q, the overall profile of Chr 3 shows that 3p is generally deleted and 3q amplified in some cell lines. They look like the inverse of one another. Furthermore, the phenomenon of amplification in 3q appears to be quite different from that in 5p. For instance, the level of gain or amplification was lower than in 5p and it did not include the entire 3q arm in all the cell lines; instead, only certain sparse regions were found altered recurrently. In fact, the average log2 ratio of MRRs at 5p was almost 2 fold times higher than those located at 3q (p<0.001, t-test; calculated from Table S1). The full arm (CaLo and HeLa) or several regions (CaSki and SiHa) of 3q were found to be amplified (Figure 6), similarly to the findings in previous reports [5], [7], [9], [19]. This could explain the lower proportion (13.4%) of deregulated genes found in 3q compared with that in 5p (33.5%). However, even in those cell lines where most of 3q was gained, the proportion of deregulated genes did not rise (CaLo) or increased modestly (HeLa). In addition, in 3q, the proportion of downregulated genes was higher than the proportion of upregulated genes, particularly in 3q26, where 8 of 11 deregulated genes were downregulated, even some of them were recurrently gained. Furthermore, similarly to 5p, deregulated genes seemed to be grouped in clusters in 3q26–29. These findings indicate that an increase in the copy number does not necessarily mean that genes located in those regions will be upregulated. It suggests that, in those entirely amplified regions, epigenetic mechanisms could be involved in gene repression. On the other hand, the increased frequency of downregulated genes with the number of amplified SNPs in the subset of genes located in MRRs, which seems to be not entirely amplified (having less than 500 SNPs; Figure 5C), supports that partial gene amplification may be a mechanism of gene silencing. This idea has been proposed theoretically [44].

The 3q26 region has been previously identified as gained or amplified in biopsies or cell lines derived from CC by using CGH or FISH [4], [6], [7], [45]. Recognized tumor genes, such as *EVI1* and *MDS1* [46], and genes associated previously with CC (*TERC*, *TNFSF10*, and *PIK3CA*) are located in this region. However, it has not been demonstrated that these genes were upregulated [5], [7], [9], [19], particularly in the same samples where the CN alterations were found. In this study, *EVI1*, *TERC*, *PIK3CA*, and *LAMP3* were neither found CN altered recurrently nor upregulated in all the cell lines studied. *TERC* was found gained in CaLo, CaSki, and HeLa but upregulated only in HeLa (data not shown). However, conclusions with these negative results from the microarrays may be too risky without the validation with different methodologies, like qPCR and qRT-PCR. Interestingly, the gene encoding for tumor necrosis factor (ligand) superfamily member 10 (TNFSF10), a protein that induces apoptosis in transformed and tumor cells, was found to be downregulated in the 4 cell lines, even though the gene was recurrently gained (MRR 3-13; Figure 6B). This gene is located in the same region as 2 other downregulated (*NLGN1* and *NAALADL2*) and 2 upregulated (*AADACL1* and *ECT2*) genes, which have not been previously associated with CC. However, the protein encoded by *ECT2* (epithelial cell transforming sequence two oncogene) is a transforming protein that is a nuclear guanine nucleotide exchange factor (GEF) and regulates RhoB-mediated cell death after DNA damage in cervical cell lines [47]. The expression of this gene is elevated with the onset of DNA synthesis and remains elevated during the G2 and M phases [48]. Increase in gene dosage by DNA amplification is a common mechanism to achieve overexpression of genes in tumors [49]. *ECT2* showed the highest fold change of the upregulated genes found at 3q; therefore, it is a good candidate for the amplified driven oncogene in 3q26 for CC.

In 1q the correlation between CN and gene expression was also very poor and similar to the figures seen in 3q. However 3 genes, IQGAP3, CENPF and PARP1, were upregulated more than 3 fold times compared with controls. The protein codify by PARP1 (Poly ADP-ribose polymerase-1) is a DNA binding protein that detects specifically DNA strand breaks generated by different genotoxic agents. Whereas activation of PARP-1 by genotoxic stimuli facilitates DNA repair and cell survival, severe DNA damage triggers different pathways of cell death, including PARP-mediated cell death [50]. Cells with BRCA1 loss of function are deficient in DNA double strand break repair thus activating PARPs whose catalytic activity is immediately stimulated by DNA strand-breaks [51]. Although in these cell lines the expression of BRCA1 and BRCA2 genes did not change (data not show), PARP1 could help these genes in that DNA reparation pathway.

### Conclusions

The overall correlation between the CN alterations and changes in gene expression was about 15% in CC cell lines. This low correlation could be related to several factors. First, most genes located in the CNAs, identified formerly in the cell lines with the 100 K microarray, were not altered in the number of copies. Second, in the genomic segments confirmed entirely amplified, like 5p, the percentage of deregulated genes was over 33%, but not all of them were upregulated. Therefore, it is clear that not all amplified genes are upregulated; instead, some of them may be repressed, possibly by epigenetic mechanisms. Third, deregulated genes were found in clusters, suggesting that, besides the segment amplification, the location in the same chromatin region may influence gene expression. Fourth, the steady rise of downregulated genes with the increase of amplified SNPs in regions CN altered discontinuously suggests that partial gene amplification could be a mechanism of silencing gene expression. Additional genes were identified up- or downregulated at 5p, 3q and 1q that could be involved in cervical carcinogenesis, particularly in apoptosis, including *CLPTM1L*, *AHRR*, *PDCD6*, and *DAP* in 5p, *TNFSF10* and *ECT2* in 3q and PARP1 in 1q.

## Materials and Methods

### Ethics Statement

The study protocol was approved by the Ethics and Scientific Committees of the Hospital General de Mexico with the approval number DIC/03/311/04/051 and was performed in accordance with the ethical standards laid down in the 1964 Declaration of Helsinki. All participants signed informed written consent forms prior to their inclusion in the study.

### Cell lines

Human cervical cancer cell lines HeLa, SiHa and CaSki were provided by Dr. Nicolás Villegas-Sepulveda [52] from the Departamento de Biomedicina Molecular, CINVESTAV-IPN, Mexico city, who purchased the cell lines in ATCC, Rockville, MD. CaLo cell line was a kind gift of Dr. Alberto Monroy-García from the Unidad de Investigación Médica en Enfermedades Oncológicas, CMNS-XXI IMSS, Mexico city, who isolated the cells [53]. All of them were maintained in RPMI 1640 medium (Life Technologies, Grand Island, NY) supplemented with 10% fetal calf serum, streptomycin, and penicillin at 37°C in a humid atmosphere containing 5% CO_2_.

### Control samples

DNA obtained from blood samples collected from 38 healthy women was used as controls for 100 K microarray analysis. Ten samples of normal cervical epithelium were used as controls for the analysis of gene expression. These samples were obtained from cervical specimens of patients undergoing hysterectomy due to myomatosis at the Gynecology Service in the Hospital General de Mexico. Patients were previously diagnosed with a normal cervix by colposcopy and cytology. Immediately after receiving a cervix fragment from the operating room, the exocervical epithelium was dissected with the aid of a stereoscopic microscope to avoid stromal cells. Then it was snap frozen in liquid nitrogen and stored at −80°C until use.

### DNA and RNA isolation

DNA was obtained with the PureLink genomic DNA kit (Invitrogen, Carlsbad, CA) and maintained at −20°C until analysis. Total RNA was extracted using the TRIzol reagent (Invitrogen, Carlsbad, CA) according to the manufacturer's instructions. The quality of RNA was confirmed by the presence of intact ribosomal RNA (28 s and 18 s bands) by using agarose-gel electrophoresis.

### GeneChip Mapping 100 K

The SNP arrays of 100 K analyze 116,204 SNPs with a mean inter-marker distance of 23.6 kb. Array experiments were performed according to the Affymetrix GeneChip Mapping 100 K standard protocols (Affymetrix Inc., Santa Clara, CA, USA). Briefly, 250 ng of DNA were digested with the appropriate restriction enzyme (XbaI or HindIII), PCR amplified, fragmented, and labeled. Microarrays were hybridized, washed, and scanned using the GeneChip 3000 scanner and Affymetrix GeneChip Command Console software. Cell intensity files (.CEL) were generated, saved, and transported to a workstation that contained Affymetrix Genotyping Console (GTC) 4.0 software.

### SNP calling

SNP calls were generated by the Bayesian Robust Linear Model with the Mahalanobis distance classifier algorithm. This algorithm performs a multiple-chip analysis that facilitates the estimation of probe effects and allele signals simultaneously and, if necessary, borrows the information of other SNPs to better predict the properties of the clusters formed by the genotypes. To maximize the accuracy of calling, the analysis was performed in a single run including the 38 controls.

### Copy number analysis

DNA copy number (CN) was calculated based on the hybridization intensity of each SNP probe and was estimated from raw signal data by the GTC 4.0. The software compares the cell lines to a reference set of normal samples. For this analysis, the protocol of unpaired samples was followed. Briefly, the parameters were set as follows: quantile normalization was performed at the perfect match probe level, followed by summarization of the signal intensity for each allele of each SNP. Genomic smoothing was set to 0.5 Mb and a 5-state hidden Markov model was applied for smoothing and segmenting CN data. The different states were defined as follows: 0 = homozygous deletion, 1 = heterozygous deletion, 2 = normal diploid, 3 = single copy gain, and 4 = amplification. Each SNP was fitted to one of the possible CN states with transition decay of 10 Mb and a threshold for SNP outlier of 1000 bp. The software output the CNCHP files that contained the estimation of CN altered SNPs in the cell lines, according to the parameters previously described. Finally, the CN altered regions (CNAs) were defined by a copy number segment reporting tool. The CNAs are the segments of DNA where a continuous succession of 2 or more CN-altered SNPs is located (see Figure S1). When the CN-altered SNPs were isolated, surrounded by non altered SNPs, they were considered CNAs of one SNP. In addition, the CN alterations were analyzed with the SVS ver 7.1 software of Golden Helix. The .CEL intensity files from the four cell lines and 38 controls were imported into the copy number analysis module of SVS. The raw intensity data from the XbaI and HindIII arrays of each sample were quantile normalized. After the normalization was performed, the log_2_ ratio was calculated using the normalized probes intensities with controls as reference. The calculated log_2_ ratio of chromosomes 1, 3 and 5 from the cell lines CaSki, HeLa, SiHa and CaLo were plotted and smoothed with the median using a window radius value of 99. Figure S2 shows that the log_2_ ratio profiles of chromosomes 1, 3 and 5 obtained with both softwares are almost identical.

### Gene expression profiling and data analysis

Gene expression profile was explored by triplicate experiments in CaLo, CaSki, HeLa, and SiHa cell lines and 10 cervical epithelium controls by using the Human Gene 1.0 ST oligonucleotide microarray (Affymetrix, Santa Clara, CA). This array contains 33,297 probe sets that correspond to approximately 20,741 genes of the human gene reference database according to UCSC Genome Browser Assembly Mar. 2006 NCBI 36/hg18, available at http://genome.ucsc.edu/. A total of 300 ng of RNA of each cell line or control sample was used for the synthesis of cDNA. This was done with SuperScript II reverse transcriptase and oligo(dT) primer, containing a T7 RNA polymerase promoter, by using the GeneChip WT cDNA synthesis kit (Affymetrix). Then *in vitro* transcription amplification was performed overnight using the GeneChip amplification kit (Affymetrix). The cRNA was random primed to include dUTP, and single-stranded DNA was fragmented with uracil DNA glycosylase followed by exonuclease 1. Fragmented DNA was then labeled using terminal deoxy-nucleotidyl transferase (TdT) and biotinylated nucleotides (GeneChip Terminal Labeling Kit; Affymetrix). A hybridization cocktail was prepared that included the labeled target DNA and control probes for hybridization. The microarrays were hybridized for 16 hours at 45°C and 60 rpm, then washed and stained with streptavidin phycoerythrin conjugate in a GeneChip Fluidics Station 450. Finally, the chips were scanned using a GeneChip Scanner 3000. Array hybridization, scanning, and image analysis were done according to the manufacturer's protocols (Affymetrix GeneChip Expression Assay manual). To assess the quality of the experiments, the following parameters were used: the expression of the exogenous polyA controls, the presence of the oligo B2 used to make grid alignments, and the values of the area under the curve (AUC) above 0.8. Only those microarrays with optimal quality controls were then analyzed. Microarrays were normalized using the RMA algorithm (robust multichip average) in the Affymetrix expression console. The values of the normalized intensity were referred to as units of intensity (UI). The identification of genes expressed differently between cell lines and controls was performed with the algorithm “Significance Analysis of Microarrays” (SAM Version 3.0, http://www.stat.stanford.edu/~tibs/SAM) by using cut-off values of fold change of ≥1.5, a general fold discovery rate (FDR) of 0%, and a local FDR of <10% [54]. The 3 experiments of each cell line or all cell-lines experiments together (n = 12) were compared with the set of controls (n = 10).

### Validation of global gene expression by real-time quantitative retro transcription PCR (qRT-PCR)

Reverse transcription of total RNA was performed using the High-Capacity cDNA Archive kit (Applied Biosystems, CA) in a total volume of 20 µL. The mix included 2 µg of RNA, 2 µL 10× RT buffer, 0.8 µL dNTPs 100 mM, 2 µL 10× RT Random Primers, 1 µL MultiScribeTM reverse transcriptase (5 U/µL), and 1 µL RNase inhibitor (2 U/µL). Reactions were incubated at 37°C for 120 min and then stored at −20°C. A set of 23 genes was used to validate gene expression in the 4 cell lines and 10 healthy cervical epithelium controls with qPCRs. The following TaqMan gene expression assays (Applied Biosystems) were used: *CCNB2* (Hs00270424_m1), *CDC2* (Hs00364293_m1), *CDC20* (Hs00415851_g1), *CDKN2A* (Hs00233365_m1), *CDKN3* (Hs00193192_m1), *CKS2* (Hs00854958_g1), *MCM2* (Hs00170472_m1), *MKI67* (Hs00606991_m1), *NUSAP1* (Hs00251213_m1), *PRC1* (Hs01597831_m1), *RFC4* (Hs00427469_m1), *TOP2A* (Hs01032127_g1), *TYMS* (Hs00426591_m1), *ZWINT* (Hs00199952_m1), *PARP1* (Hs00242302_m1), *NAALADL2* (Hs00822484_m1), *POLD1* (Hs00172491_m1), CLPTM1L (Hs00363947_m1), TRIO (Hs00179276_m1), TNFSF10 (Hs00921974_m1), NLGN1 (Hs00208784_m1), ECT2 (Hs00216455_m1) and *RFC5* (Hs00738859_m1). *GAPDH* (Hs02758991_g1) and *BETA ACTIN* (Hs01064292_g1) were used as controls. Genes located in 1q (PARP1), 3q (MCM2, TNFSF10, ECT2, NLGN1, NAALADL2, RFC4) and 5p (TRIO, CLPTM1L) were selected according to the specific Chr analyzed in this study. The rest of genes are located in other chromosomes and were selected to validate gene expression because most of them ranked throughout the first 100 places of de-regulated genes in cell lines (Table S4). The experiments were run in duplicate in a final volume of 20 µL including 200 ng of cDNA template, 10 µL of 2× TaqMan Universal PCR Master Mix (Applied Biosystems, CA), 1 µL of 20× TaqMan Gene Expression Assay, and 7 µL of RNase-free water. The cycling program was run in a Rotor-Gene (Corbett Research, Sydney, Australia) and set as follows: PCR initial activation step at 50°C for 2 min followed by 95°C for 10 min, then 40 cycles of melting at 95°C for 15 s and annealing/extension at 60°C for 1 min. Measurement of gene expression was based on a relative standard curve constructed from a 10-fold serially diluted pool of the 4 cell-line cDNAs ranging from 500 to 0.05 ng/µL. The expression of target genes was normalized in each cell line and control sample to the median intensity of the internal references by using a method previously described in detail [55]. The values of the normalized intensity were measured in ng/µL. The fold-change expression was calculated by dividing the normalized intensity of each cell line by the average normalized intensity of the control samples. The statistical difference between each cell line and the set of controls was measured with a Mann–Whitney non-parametric test. The level of correlation between the microarray results and qRT-PCR data was measured with the Pearson's correlation coefficient.

### Fluorescent in situ hybridization (FISH)

Interphase and metaphase preparations of CaSki, HeLa, and SiHa were obtained according to standard procedures [56]. Double-color FISH experiments were performed to determinate the copy numbers of 3 regions, i.e., 5p15.3, 5p15.2, and 3q26. For 5p, 2 cocktails of probes were used (Abbott Laboratories. Abbott Park, Illinois, USA). One included a target probe located at 5p15.3 (C84c11/T3, labeled with SpectrumGreen) and a control probe (D5S2907, labeled with SpectrumOrange) located at 5q35.3, and the other included a target probe located at 5p15.2 (LSI D5S23, D5S721, labeled with SpectrumGreen) and a control probe (LSI *EGR1*, labeled with SpectrumOrange) located at 5q31.1–31.3. For 3q, 1 cocktail of probes (KBI-10110; Kreatech Diagnostics, Amsterdam) was used, which included a target probe (*hTERC* locus) located at 3q26.2, labeled with PlatinumBright 550 (red), and a centromeric 3q11 probe, labeled with PlatinumBright 595 (green), as the control. Slide preparation, DNA hybridization, and post-hybridization washes were carried out using standard methods described in the manual. At least 20 cells were analyzed using direct microscopic visualization and digital-imaging analysis to verify number of signals and probe location. The copy number changes were measured by calculating the ratio between the average number of signals of target and control probes.

### Validation of PARP1 copy number by real-time quantitative PCR (qPCR)

The number of copies of PARP1 gene was calculated in the four cell lines using 10 samples of lymphocytes DNA as reference control. Experiments were run by triplicates and performed on a Rotor-Gene 6000 Corbett detection system (Corbett Life Science), using the TaqMan® Assays. The copy number of PARP1 and RNase P genes was determined together in a single tube. We used 6.4 ng genomic DNA in 20 µL of reaction mixture consisting of TaqMan® Genotyping Master Mix (Applied Biosystems), TaqMan® Copy Number Reference Assay RNaseP (4401631) and TaqMan® Copy Number Assay PARP1 (Hs 05725717_cn). The cycling conditions consisted of an initial hold of 95°C for 10 minutes, followed by 40 cycles of 95°C for 15 seconds, and 60°C for 60 seconds. Quantification was performed using both the relative standard curve method and the comparative C_T_ method. The values of PARP1 were normalized in all samples with the values of RNase P. The copy number was calculated by dividing the normalized values of PARP1 of each cell line between the median values of the control samples and then multiplied by 2.

### Gene ontology classification analysis

The Database for Annotation, Visualization, and Integrated Discovery (DAVID) functional annotation tool (http://david.abcc.ncifcrf.gov) [57], [58] and the Ingenuity Pathway Analysis (IPA; Ingenuity® Systems, www.ingenuity.com) were used to classify the gene list obtained from high-throughput platforms. Genes were classified using functional annotation clustering with consideration of the gene ontology biological processes. Classification stringency was set at the maximum level.

### Gene annotation and data integration

The physical position of SNPs, DNA segments, and genes was mapped according to UCSC Genome Browser Assembly Mar. 2006 NCBI 36/hg18, available at http://genome.ucsc.edu/. The CNAs of cell lines were aligned according to the position in the genome, and minimal recurrent regions (MRRs) common to all 4 cell lines were identified (Figure S1). The analysis of SNPs, CNAs, MRRs, and genes and the alignments of all of them, according to the position in the genome, were performed with Access 2010 (Microsoft Inc.). To identify the arms, cytobands or MRRs with the higher alterations, an enrichment analysis, based on a chi squared test, was performed. The frequencies of the CN-altered SNPs, CN-altered genes or deregulated genes in each of those regions were compared with the frequencies found in the whole genome of cell lines. A chi square or Fisher exact test, as appropriate, were used to evaluate the statistical significance of the differences. In addition, a Parametric Gene Set Enrichment Analysis (PAGE) procedure was performed to evaluate the enrichment of deregulated genes in chromosomal arms and cytobands [59]. This procedure is based on the calculation of the Z score, which takes into account the number of deregulated genes and the fold change (FC).

The mean (μ) and standard deviation (δ) of fold change of total deregulated genes (3,122) and the mean fold change (Sm) of deregulated genes (m) located in a given arm or cytobands were calculated. Then, the Z score was calculated as Z = (Sm−μ)*m^1/2^/δ. Microsoft Excel was used to calculate p-values from Z scores. Gene sets with less than 10 genes were discarded. Two tails p-values<0.05 were considered statistically significant [59]. For 1q, 3q and 5p the genes were ordered according to its position in the chromosome and the fold change graphed. The identification of clusters of 2 or more contiguous deregulated genes were identified by simple inspection and to test whether this distribution was statistically significant from a random distribution, a chi square test was used. The raw microarray data of both SNPs and expression is MIAME compliant and has been deposited in a MIAME compliant database (GEO, http://www.ncbi.nih.gov/geo/) under the accession number GSE29245. Sigma Stat and SSPS softwares were used for statistical comparisons among the groups.

## Supporting Information

Figure S1 **Construction of CNAs and MRRs.** An amplified CNA (red bar) is shown in panel A and included the segment of DNA where the continuous succession of 10 amplified SNPs (red triangles) are located .CNAs of the four cell lines are aligned according to the position in the genome, and a minimal recurrent regions (MRR) common to all 4 cell lines is identified (panel B). Genes are aligned according to the position in the genome. Genes in red are those located into the CNAs or MRR. The triangles and genes in white, surrounding the amplified CNA, are SNPs and genes with CN = 2. Notice that genes inside the CNA can be located in sub regions with or without SNPs (panel A).
(TIF)

Figure S2 **Log2 ratio profiles of Chr 1q, 3q and 5p.** The figure shows that the log_2_ ratio profiles of chromosomes 1, 3 and 5 obtained with both softwares, the Command Console of Affymetrix and the SVS of Golden Helix.
(TIF)

Table S1 **Minimal recurrent regions.** a. Combined = deleted in some cell lines and gained in others. b. The value is the average Log2 ratio of all the SNPs including in the minimal recurrent region of the four cell lines. c. The value is the average of all the SNPs of the minimal recurrent region. Values>1.3 correspond to p<0.05 and indicate that the difference in the signal intensity between the cell lines and the control group is statistically significant (t-test). d. The percent was calculated in relation to the number of genes per MRR (eleventh column).
(XLSX)

Table S2 **Comparison between copy number and genes expression by cytoband.** a. Chi square test. b. Z test. c. Include cytobands with more than 5.8% of altered SNPs and p-value<0.05 (chi square) compared with the whole genome. The cytobands shadowed in blue were those with more than 30% of CN altered SNPs. d. Include cytobands with more than 15.1% of deregulated genes and p-value<0.05 (chi square) compared with the whole genome or Z score >1.96 or <−1.96. Z score was calculated as part of the Parametric Gene Set Enrichment Analysis.
(XLSX)

Table S3 **Genes identified in the minimal recurrent regions.** a. Combined = genes deleted in some cell lines and gained in others. b. NE = not explored, UN = unchanged, UP = upregulated, DOWN = downregulated.
(XLSX)

Table S4 **Genes expressed differently in cell lines compared with normal cervical epithelium^a^.** a. This list of genes was obtained when the 4 cell lines, each by triplicate, were compared together against the control sample (n = 10) using the SAM method. b. Fold Change was obtained dividing the normalized signals of cell lines/healthy cervical controls.
(XLSX)
