# Supplementary figures and images for: Amplified Genes May Be Overexpressed, Unchanged, or Downregulated in Cervical Cancer Cell Lines
Source: PLoS One. 2012 Mar 7;7(3):e32667. doi: 10.1371/journal.pone.0032667 (PMC3296745; doi:10.1371/journal.pone.0032667)

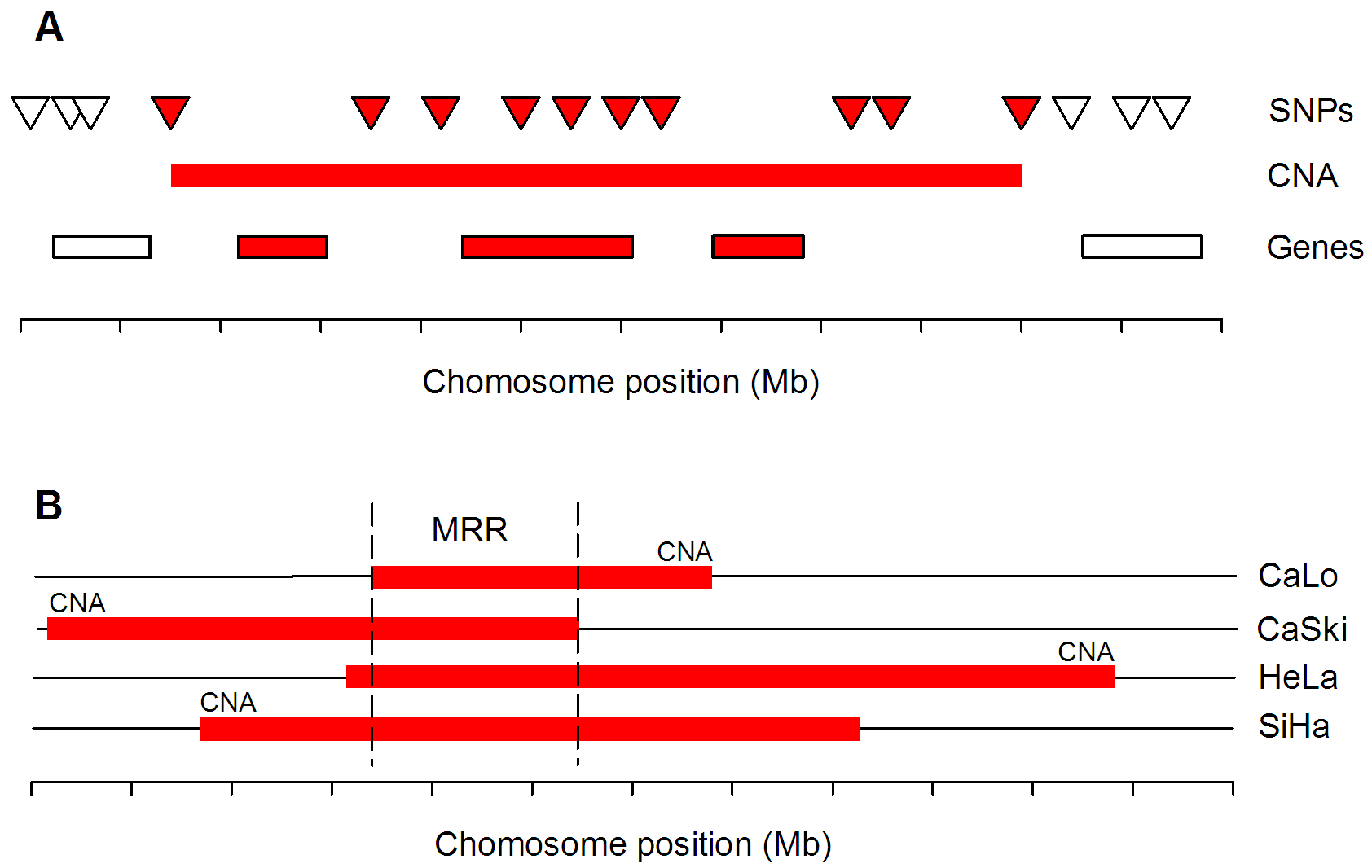

Supplement: Figure S1 — Construction of CNAs and MRRs. An amplified CNA (red bar) is shown in panel A and included the segment of DNA where the continuous succession of 10 amplified SNPs (red triangles) are located .CNAs of the four cell lines are aligned according to the position in the genome, and a minimal recurrent regions (MRR) common to all 4 cell lines is identified (panel B). Genes are aligned according to the position in the genome. Genes in red are those located into the CNAs or MRR. The triangles and genes in white, surrounding the amplified CNA, are SNPs and genes with CN = 2. Notice that genes inside the CNA can be located in sub regions with or without SNPs (panel A). (TIF) [file pone.0032667.s001.tif]

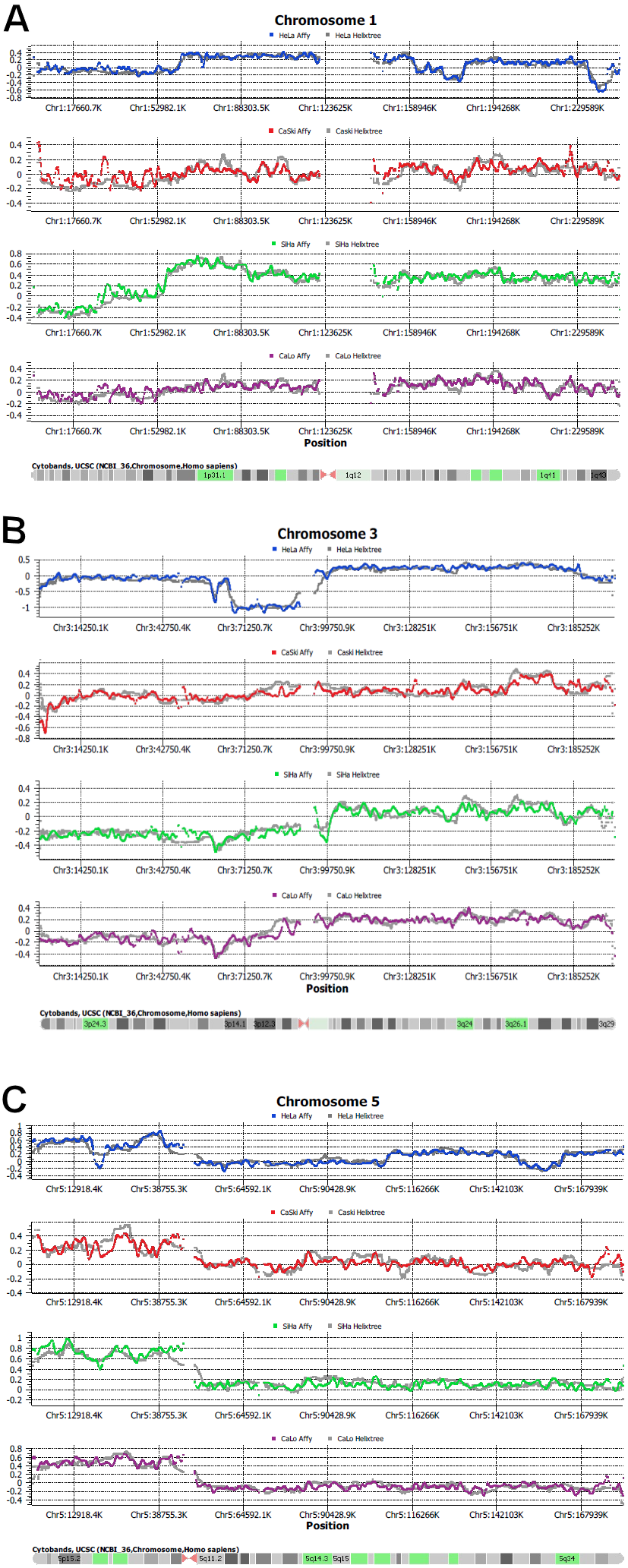

Supplement: Figure S2 — Log2 ratio profiles of Chr 1q, 3q and 5p. The figure shows that the log2 ratio profiles of chromosomes 1, 3 and 5 obtained with both softwares, the Command Console of Affymetrix and the SVS of Golden Helix. (TIF) [file pone.0032667.s002.tif]
